# Case Report: Rare triple-line chromosome 9 mosaicism (47,XX,+del(9)(q13)/47,XX,+9/46,XX) associated with severe neurodevelopmental impairment and congenital anomalies

**DOI:** 10.3389/fgene.2026.1769931

**Published:** 2026-04-07

**Authors:** Andreja Zagorac, Mirjana Miksić, Boris Zagradišnik, Bojan Korpar, Nadja Kokalj Vokač

**Affiliations:** 1 Clinical Institute for Genetic Diagnostics, University Medical Centre Maribor, Maribor, Slovenia; 2 Department of Perinatology, University Medical Centre Maribor, Maribor, Slovenia

**Keywords:** chromosome 9 mosaicism, congenital heart defects, craniofacial dysmorphism, neurodevelopmental delay, triple-line mosaicism

## Abstract

**Background:**

Chromosome 9 abnormalities are rare. Full trisomy 9 is mostly incompatible with postnatal survival, whereas mosaic trisomy 9 permits survival with highly variable clinical manifestations. Triple-line mosaicism involving both full trisomy 9 and structural chromosome 9 abnormalities is exceptionally rare.

**Case Presentation:**

A 1-year-old girl, conceived via IVF, had normal nuchal translucency and NIPT. Prenatal ultrasound showed a single umbilical artery, lower abdominal cyst, borderline ventriculomegaly at 28 weeks, and late polyhydramnios. She was born at 40 weeks, 3,430 g, 49 cm, head circumference 36 cm, Apgar 8/9/9, requiring mild oxygen supplementation. At age 1 year, she exhibited dysmorphic facial features (prominent forehead, hypertelorism, upslanting palpebral fissures, short philtrum, low-set mildly dysplastic ears, retrognathia), bilateral ovarian cysts, minimal clinodactyly of the right fifth finger, palmar and plantar crease abnormalities, hemodynamically significant patent ductus arteriosus (PDA), patent foramen ovale (PFO), previously closed muscular ventricular septal defect (VSD), severe hypotonia, global developmental delay, convergent strabismus, and feeding difficulties requiring gastrostomy. Cranial ultrasound revealed ventriculomegaly, grade II intraventricular hemorrhage, slightly thin corpus callosum, and altered posterior cranial fossa. Postnatal genetic evaluation, including array CGH (comparative genomic hybridization), conventional karyotyping, FISH (fluorescent in situ hybridization), and QF-PCR (Quantitative Fluorescence Polymerase Chain Reaction), identified a complex mosaicism comprising three cell lines: 47,XX,+del(9)(q13)[60%]/47,XX,+9[20%]/46,XX[20%].

**Conclusion:**

This case expands the phenotypic spectrum of chromosome 9 mosaicism and highlights the diagnostic value of multimodal genomic analysis for accurate detection and counselling.

## 1 Introduction

Trisomy 9 is a rare chromosomal condition. Complete, non-mosaic trisomy 9 is usually incompatible with postnatal survival, whereas mosaic trisomy 9 permits survival but presents highly heterogeneous phenotypes, depending on the proportion and tissue distribution of abnormal cells (Arnold et al., 1995; BC Children’s Hospital Research Institute, 2025). Survivors may display craniofacial dysmorphism, skeletal anomalies, congenital heart defects, genitourinary malformations, growth retardation, and significant neurologic impairment (BC Children’s Hospital Research Institute, 2025).

Partial duplications of chromosome 9, especially 9p, represent a separate group of rare chromosomal imbalances. Individuals with 9p duplication syndrome show distinctive facial features, developmental delay, intellectual disability, and congenital anomalies affecting central nervous system (CNS) and cardiovascular systems (Pejčić et al., 2018; Park et al., 2020). Phenotypic overlap between mosaic trisomy 9 and 9p duplication complicates genotype–phenotype correlation.

Mosaic cases combining both numerical (full trisomy) and structural (partial duplication/deletion) abnormalities of chromosome 9 are exceedingly rare (Sherer et al., 1992). Such cases likely arise via postzygotic mitotic errors and/or sequential chromosomal rearrangements, resulting in clonal diversification. Tissue-specific mosaic distribution strongly influences phenotype. Because of this variability, accurate genotype–phenotype correlation remains challenging, and longitudinal clinical data are scarce (BC Children’s Hospital Research Institute, 2025; Sherer et al., 1992).

High-resolution genomic technologies have highlighted limitations of single-method diagnostics. Chromosomal microarray (CMA/aCGH) can detect copy-number imbalances but may underestimate mosaicism. FISH and QF-PCR are required to reveal minor or structural abnormal clones (Ma et al., 2023; Yang et al., 2025). Multimodal cytogenetic evaluation is therefore essential when clinical features suggest mosaicism beyond array detection.

Here, we report a 1-year-old girl with three coexisting chromosome 9 cell lines: 47,XX,+del(9)(q13)/47,XX,+9/46,XX, presenting with severe neurodevelopmental impairment, feeding difficulties, dysmorphism, ovarian cysts, and congenital cardiac anomalies. We provide comprehensive clinical, radiologic, and molecular characterization.

## 2 Case description

The patient is a one-year-old girl, the first child of her family, conceived via *in vitro* fertilization (IVF) after 12 months of unexplained infertility, to a 38 years mother and a 40 years old father. Her prenatal course was generally unremarkable, with normal first-trimester screening and non-invasive prenatal testing (NIPT). Prenatal ultrasound revealed a single umbilical artery, a cyst in the lower abdomen, and borderline ventriculomegaly at 28 weeks. Polyhydramnios developed in the late third trimester, prompting closer monitoring. She was delivered at 40 weeks gestation, weighing 3,430 g (≈50th percentile), 49 cm in length (≈10th percentile), and with a head circumference of 36 cm (≈90th percentile). Apgar scores were 8, 9, and 9 at 1, 5, and 10 min, respectively, and mild oxygen supplementation was required.

At 1 year of age, the patient displayed characteristic craniofacial dysmorphism, including a prominent forehead, hypertelorism, upward-slanting palpebral fissures, a short philtrum, retrognathia, and low-set, mildly dysplastic ears. Cardiac examination revealed a symmetrical chest with a regular rhythm; a previously hemodynamically significant patent ductus arteriosus had been closed percutaneously, a muscular ventricular septal defect had spontaneously closed, and a patent foramen ovale persisted. Abdominal and genitourinary examination was unremarkable, though ultrasonography identified bilateral ovarian cysts. Hands exhibited a suggestive four-finger crease, and the feet showed slight alterations in plantar creases, with minimal clinodactyly of the right fifth finger. Neurologically, eye contact was brief, bilateral convergent strabismus was present, and generalized hypotonia was noted. Spontaneous motor activity was limited, and she was unable to sit independently. Persistent feeding difficulties necessitated a gastrostomy tube. Cranial ultrasonography revealed ventriculomegaly, grade II intraventricular hemorrhage, a slightly thin corpus callosum, and alterations of the posterior cranial fossa.

The patient remains under ongoing multidisciplinary follow-up with pediatric cardiologists, neurologists, endocrinologists, gastroenterologists, and developmental specialists.

## 3 Genetic findings

During pregnancy, routine prenatal screening, including first-trimester nuchal translucency measurement and NIPT, did not reveal an increased risk for common aneuploidies. Ultrasound examinations identified a single umbilical artery, a cyst in the lower abdomen, borderline ventriculomegaly at 28 weeks, and polyhydramnios in late pregnancy. No invasive prenatal genetic testing was performed with normal result.

**FIGURE 1:**
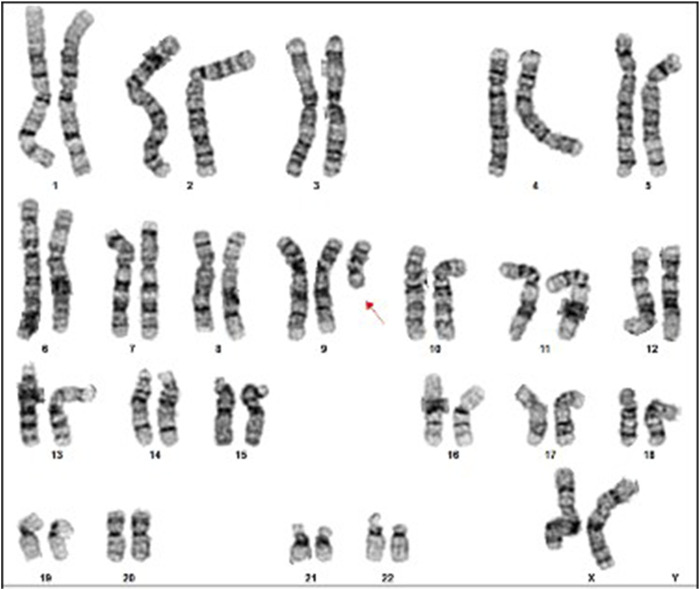
Karyotype of the majority of the cells: 47,XX,+del(9)(q13).

**FIGURE 2:**
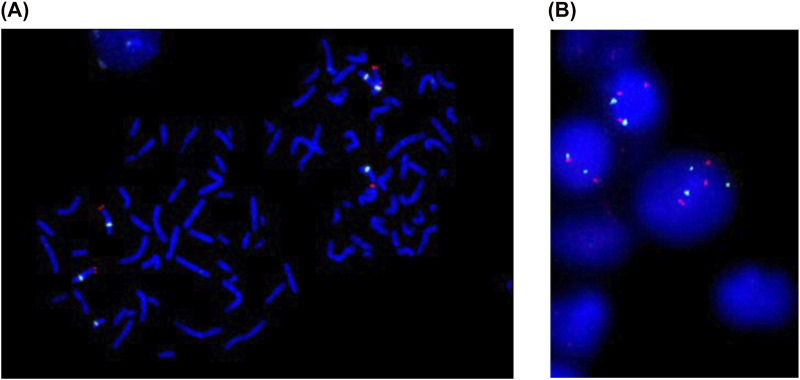
**(A)** Metaphase FISH using CEP9 (aqua) and 9qter (red) DNA probes (Vysis-Abbott) showing metaphase with additional 9p (left) and full trisomy 9 (right). **(B)** Interphase FISH using 9p21 (red) and 9q21 (green) DNA probes (Kreatech-Leica) showing all three cell lines: normal (left), +9p (middle), +9 (right).

**FIGURE 3:**
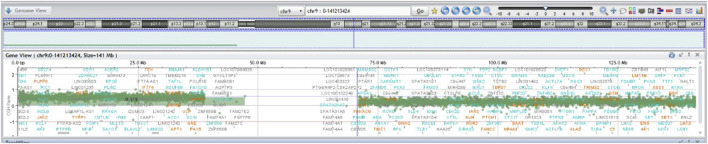
SurePrint G3 Custom CGH Array 4 × 180K, CytoGenomics 5.0.0.38 (Agilent Technologies): arr(9p)x3[0.8],(9q)x3[0.2].

Postnatally, genetic evaluation at 1 year of age included conventional karyotyping and FISH on cells isolated from peripheral blood, and isolated DNA for array CGH, and QF-PCR analysis. On 200 metaphases examined: 100 using GTG banding and 100 using FISH (CEP9(aqua)+9qter(red)): 160 metaphases reveled the karyotype 47,XX,+del(9)(q13), 2 metaphases: 47,XX,+9 and 38 metaphases were normal: 46,XX (Figures 1, 2A). Additional 400 interphase were examined by FISH, 240 nuclei showed + del(9)(q13), 80 cells: +9 and 80 cells were normal (Figure 2B). Array CGH demonstrated a gain of the entire short arm of chromosome 9 in ∼80% of cells and a gain of the long arm in ∼20%: arr(9p)x3[0.8],(9q)x3[0.2] (Figure 3). QF-PCR confirmed a third cell line representing full trisomy 9. The final mosaic karyotype according to ISCN 2024:

mos 47,XX,+del(9)(q13)[160]/47,XX,+9[2]/46,XX[38].nuc ish(CDKN2Ax3,9q21x2)[240/400]/(CDKN2A,9q21)x3[80/400].arr (9p)x3[0.8],(9q)x3[0.2].

Both parents were evaluated for the presence of low-level mosaicism using FISH with CEP 9 DNA probes. Interphase FISH analysis and metaphase karyotyping were performed on cultured peripheral blood lymphocytes. A total of 200 interphase nuclei and 100 metaphase cells were examined for each individual. Normal results were obtained.

## 4 Follow-up and prognosis

Since birth, the patient has required close multidisciplinary monitoring. Neurodevelopmental impairment remains severe, with persistent hypotonia and limited motor activity. Ophthalmologic evaluation revealed bilateral convergent strabismus. Cardiac anomalies have been managed, including closure of PDA and spontaneous resolution of VSD, with ongoing monitoring of PFO. Feeding difficulties persist and are managed via gastrostomy. Early intervention, physical and occupational therapy, and nutritional support are essential. Given the complex chromosomal mosaicism, long-term neurodevelopmental prognosis remains guarded.

Prognosis in mosaic trisomy 9 spectrum disorders is highly variable and depends primarily on (1) the proportion of full trisomy 9 cells, (2) the presence of major organ malformations, and (3) the degree of central nervous system involvement. In contrast to complete trisomy 9—which is frequently associated with perinatal lethality—long-term survival has been documented in mosaic cases, particularly when a substantial euploid cell line is present. Based on previously reported mosaic trisomy 9 cases, survival into childhood and adolescence is possible, particularly in individuals without major organ system failure. The prognosis for our patient appears favorable for survival, guarded for neurodevelopmental outcome and dependent on early supportive interventions. Her phenotype suggests an intermediate severity within the mosaic trisomy 9 spectrum. The presence of a normal cell line likely mitigates the lethal potential while central nervous system involvement remains the principal determinant of long-term functional outcome (Li et al., 2021).

## 5 Discussion

Mosaic trisomy 9 exhibits substantial clinical heterogeneity, ranging from mild dysmorphic features to severe multi-system involvement with profound developmental impairment (Arnold et al., 1995; BC Children’s Hospital Research Institute, 2025). Craniofacial dysmorphisms—prominent forehead, hypertelorism, upslanting palpebral fissures, short philtrum, retrognathia, and low-set or dysplastic ears—are consistent and were present in our patient (Pejčić et al., 2018; Park et al., 2020).

Cardiovascular involvement is frequent, including PDA, VSD, ASD, and other structural anomalies (Pejčić et al., 2018; Sherer et al., 1992). Our patient presented with a PDA, muscular VSD (spontaneously closed), and PFO, consistent with previous reports (Pejčić et al., 2018; Ma et al., 2023). CNS anomalies, such as ventriculomegaly, intraventricular hemorrhage, corpus callosum thinning, and posterior fossa alterations, correspond with severe global developmental delay and hypotonia (Park et al., 2020; Yang et al., 2025). The proportion of abnormal cells in the CNS may exceed that in peripheral blood, illustrating the limitations of blood-based cytogenetic assessments (Arnold et al., 2002; Vermeesch et al., 2005).

Genitourinary anomalies are sporadically reported in 9p duplication and trisomy 9 mosaicism (Park et al., 2020; Gu et al., 2021). Our patient had bilateral ovarian cysts, expanding the phenotypic spectrum.

In our patient with several clinical features, including typical craniofacial dysmorphism, ventriculomegaly, hypotonia, developmental delay, mild limb anomalies, and congenital heart defects, specifically patent ductus arteriosus (PDA) and ventricular septal defect (VSD), at the same time, several of the more severe anomalies commonly reported in mosaic trisomy 9 are not present. These include major limb malformations, severe renal anomalies, clefting, complex congenital heart defects, holoprosencephaly or other severe brain malformations, and seizures (Pejčić et al., 2018). This relatively milder structural presentation is consistent with mosaicism, meaning the presence of a normal cell line. It may also indicate a lower proportion of cells with full trisomy 9 or a tissue-specific distribution of abnormal cell lines. Mosaic trisomy 9 is known to have one of the broadest phenotypic spectra among autosomal trisomies. Based on the current clinical findings, this patient appears to fall toward the moderate end of the severity spectrum rather than the severe or lethal end.

*In vitro* fertilization (IVF) has been raised regarding a possible increase in congenital malformations and inborn errors among assisted reproductive technologies (ART) conceived offspring. The absolute risk increase is small, and the majority of ART-conceived children are healthy. Although ART is associated with a slightly elevated relative risk of congenital malformations and rare imprinting disorders, the overall prognosis for ART-conceived children remains favorable. The increased risk of congenital malformations discussed in ART is generally multifactorial and modest, whereas partial or complete trisomy 9 mosaicism represents a specific chromosomal abnormality with intrinsically high malformation risk, independent of ART itself (Viotti, 2020; Hansen et al., 2013; Davies et al., 2012).

The triple-line mosaicism likely resulted from early postzygotic mitotic nondisjunction followed by structural rearrangement. A normal cell line in mosaic trisomy 9 can arise through trisomic rescue, and this mechanism creates a theoretical and biologically plausible risk of uniparental disomy (UPD) 9. Parents refused additional molecular testing that would explain the origin of chromosomes 9 in the girl. Even minority abnormal cell populations may impact phenotype, especially in vital organs (BC Children’s Hospital Research Institute, 2025; Sherer et al., 1992; Arnold et al., 2002). Diagnostic challenges necessitate a multimodal approach. CMA/aCGH detects copy-number variations but may underestimate low-level mosaicism; karyotyping identifies structural rearrangements; FISH and QF-PCR allow detection and quantification of minor abnormal clones (Ma et al., 2023; Yang et al., 2025). Discrepancies between methods are common when mosaicism is tissue-limited or low-level (Ma et al., 2023; Yang et al., 2025; Vermeesch et al., 2005). Genotype-phenotype correlation remains challenging.

This case underscores the importance of multidisciplinary follow-up for cardiac, neurologic, endocrine, gastroenterologic, and developmental support. Early interventions may improve quality of life, though neurodevelopmental outcomes remain guarded in severe mosaic cases (Arnold et al., 1995; Pejčić et al., 2018; Park et al., 2020). Reporting such rare cases enhances genotype-phenotype correlations, informs prenatal counseling, and guides management strategies (BC Children’s Hospital Research Institute, 2025; Pejčić et al., 2018; Park et al., 2020).

## Data Availability

The raw data supporting the conclusions of this article will be made available by the authors, without undue reservation.
